# Inhibitory effect of phytochemicals towards SARS-CoV-2 papain like protease (PLpro) proteolytic and deubiquitinase activity

**DOI:** 10.3389/fchem.2022.1100460

**Published:** 2023-01-12

**Authors:** Anasha Kawall, Devin S. M. Lewis, Avini Sharma, Krishna Chavada, Rahul Deshmukh, Srujana Rayalam, Vicky Mody, Shashidharamurthy Taval

**Affiliations:** ^1^ Department of Biomedical Sciences, Philadelphia College of Osteopathic Medicine—Georgia Campus, Suwanee, GA, United States; ^2^ Division of Research, Philadelphia College of Osteopathic Medicine—Georgia Campus, Suwanee, GA, United States; ^3^ Department of Pharmaceutical Sciences, School of Pharmacy, Philadelphia College of Osteopathic Medicine—Georgia Campus, Suwanee, GA, United States; ^4^ Rosalind Franklin University of Medicine and Science, North Chicago, IL, United States

**Keywords:** SARS-CoV-2, PLpro, replication, phytochemicals, natural compounds

## Abstract

Recent studies have shown that RNA-dependent RNA polymerase (RdRp), 3-chymotrypsin-like protease (3CLpro), and papain-like protease (PLpro) are necessary for SARS-CoV-2 replication. Among these three enzymes, PLpro exhibits both proteolytic and deubiquitinase (DUB) activity and is responsible for disrupting the host’s innate immune response against SARS-CoV-2. Because of this unique property of PLpro, we investigated the inhibitory effects of phytochemicals on the SARS-CoV-2 PLpro enzyme. Our data indicates that the phytochemicals such as catechin, epigallocatechin gallate (EGCG), mangiferin, myricetin, rutin, and theaflavin exhibited inhibitory activity with IC_50_ values of 14.2, 128.4, 95.3, 12.1, and 43.4, and 7.3 μM, respectively, towards PLpro proteolytic activity. However, the IC_50_ values of quercetin, oleuropein, and γ-mangostin are ambiguous. We observed that EGCG, mangiferin, myricetin, oleuropein, rutin, and theaflavin have also inhibited the DUB activity with IC_50_ values of 44.7, 104.3, 29.2, 131.5, 61.7, and 13.2 μM, respectively. Mechanistically, the ligand-protein interaction structural modeling suggests that mangiferin, EGCG, theaflavin, and oleuropein shows that these four ligands interact with Glu^167^, and Tyr^268^, however mangiferin and oleuropein showed very weak interaction with Glu^167^ as compared to EGCG, and theaflavin which reflects their low IC_50_ values for DUB activity. Our data indicate that the phytochemicals mentioned above inhibit the proteolytic and DUB activity of SARS-CoV-2 PLpro, thus preventing viral replication and promoting host innate immune response. However, the therapeutic potential of these phytochemicals needs to be validated by pre-clinical and clinical studies.

## Graphical Abstract

**Figure:**
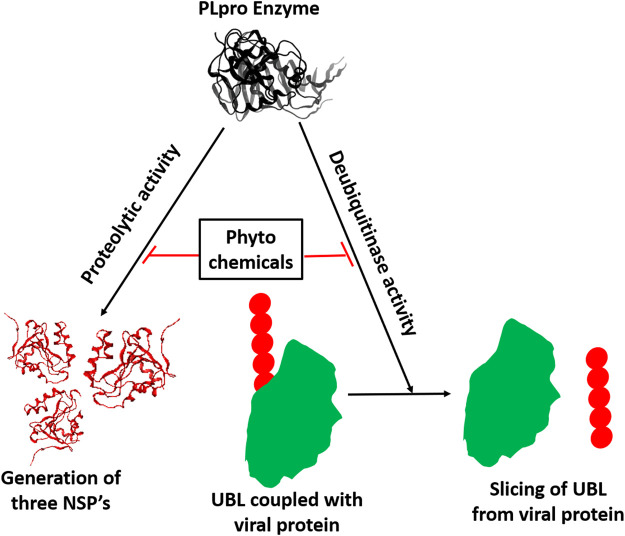
Inhibition of proteolytic and deubiquitinase activity of SARS-CoV-2 PLpro enzyme by phytochemicals.

## Introduction

The emergence of severe acute respiratory syndrome coronavirus 2 (SARS-CoV-2) resulted in the coronavirus disease 19 (COVID-19) pandemic that claimed millions of lives globally (https://coronavirus.jhu.edu/). SARS-CoV-2 belongs to β-CoVs family and has single-stranded RNA as a source of genetic material (Ul Qamar et al., 2020). The virus’s life cycle begins with its spike proteins attaching to the host cells’ angiotensin-converting enzyme 2 (ACE2) receptor (Astuti and Ysrafil, 2020). After attachment, the viral envelope undergoes membrane fusion with the host cell membrane, which permits the release of the viral genome into the host cell’s cytoplasm (Jackson et al., 2021). The viral genome (+ssRNA) uses the host’s ribosomes to translate a polypeptide chain (PP) of approximately 800 kDa ^11^. Two proteases encoded by the viral genome, papain-like proteases (PLpro) and 3-chymotrypsin-like protease (3CLpro), auto-cleave the newly generated PP chain to generate 16 non-structural proteins (NSPs) required for the viral replication (Mody et al., 2021).

Along with protease activity, SARS-CoV-2 PLpro exhibits deubiquitination (DUB) activity (Mahmoudvand and Shokri, 2021). Ubiquitination refers to the attachment of ubiquitin (UB) and ubiquitin-like proteins (UBL) to the cellular proteins that need to be degraded by the host proteasomal complex in the cytosol. Ubiquitination also plays a vital role in degrading the foreign proteins such as viral proteins upon infection to prevent viral propagation (Kikkert, 2020); thus, SARS-CoV-2 PLpro’s DUB activity disrupts the host’s antiviral immune response. Upon viral infection, innate immune cells produce Type-I interferon (IFN-α/β), inducing interferon-sensitive gene-15 (ISG-15). The upregulated ISG-15 protein conjugates with multiple signaling molecules, such as JAK, STAT, and IRF-3, through ISGylation to promote the Type-I IFN induced antiviral function (Jeon et al., 2010; Ratia et al., 2014; McClain and Vabret, 2020). It has been shown that SARS-CoV-2 PLpro mediates de-ISGylation of ISG-15 to the host signaling molecules that lead to the inhibition of the host antiviral innate immune response (McClain and Vabret, 2020; Shin et al., 2020). Thus, the SARS-CoV-2 PLpro’s DUB activity impairs the primary interferon-mediated antiviral response, which is the main feature of COVID-19 (Yang et al., 2021). Numerous reports suggest that the SARS-CoV-2 mediated mortality is caused by the pro-inflammatory cytokine storm (Hojyo et al., 2020). The causation of the pro-inflammatory cytokine storm typical in SARS-CoV-2 infection may be due to the impaired interferon-mediated antiviral response. Thus, PLpro serves as a drug target to inhibit viral replication and suppress the cytokine storm during SARS-CoV-2 infection.

The effective measures, such as vaccines (Tomalka et al., 2022) and small-molecule inhibitors (Wang and Yang, 2020; Wang and Yang, 2022a; Calleja et al., 2022; Lv et al., 2022) are greatly needed to reduce SARS-CoV-2 transmission. However, promising drugs still do not exist (Wang et al., 2022a; Wang and Yang, 2022b; Rubin, 2022). As an indispensable resource, phytochemicals (Beura et al., 2021; Kumar et al., 2021; Wang and Yang, 2021; Wang et al., 2022b) have demonstrated potential value in countering SARS-CoV-2 infection. Phytochemicals have been used as natural antiviral compounds that can inhibit viral replication or viral entry (Cheng et al., 2006; Lin et al., 2014; Chakravarti et al., 2021; Srinivasan et al., 2022). They are derived from plants, vegetables, fruits, tea, and red wine and are used in traditional medicines. Phytochemicals were shown to inhibit viral replication, RNA synthesis, viral protein synthesis, and block viral attachment to the host cell (Cheng et al., 2006; Lin et al., 2014; Chakravarti et al., 2021; Srinivasan et al., 2022). In the present study, we have investigated the inhibitory effect of several commercially available phytochemicals against the PLpro enzymatic activity assay. Some of the phytochemicals such as myricetin (extracted from nuts, berries, and red wine), theaflavin (extracted from black tea), mangiferin (extracted from mangoes), oleuropein (extracted from olives), EGCG (extracted from green tea) and rutin (extracted from buckwheat) exhibited more than 50% inhibition activity in SARS-CoV-2 PLpro enzymatic activity. These results suggest their potential benefit in preventing the replication of the SARS-CoV-2.

## Materials and methods

### Reagents and phytochemicals

Molecular biology grade DMSO was from Sigma-Aldrich (St. Louis, MO, United States). Sterile PBS was purchased from ThermoFisher Scientific (Waltham, MA, United States). Recombinant full-length untagged PLpro with His-Tag (SARS-CoV-2), assay buffers, inhibitors, fluorescently labeled substrates, and deubiquitinase substrates were from BPS Biosciences (San Diego, CA, United States). The phytochemicals were purchased from Sigma-Aldrich (St. Louis, MO), MedChemExpress (Princeton, NJ), and Cayman Chemicals (Ann Arbor, MI). The names of the phytochemicals, manufacturer names, and catalog numbers are listed in Supplementary Table S1.

### High-throughput screening enzymatic assays

Phytochemicals stock (8 mM) was prepared using either DMSO or PBS. Each phytochemical working stock (250 and 500 µM) was prepared in PBS and used for *in vitro* enzymatic assay. PLpro proteolytic and DUB assay were performed as described previously (Lewis et al., 2022). Briefly, 0.4 ng/μL of PLpro in 30 µL of assay buffer was pre-incubated with the 10 µL of 250 µM phytochemicals for 1 hour. The enzymatic reaction was initiated by adding 10 µL of 250 µM fluorescently labeled substrate. The deubiquitinase assay was initiated using PLpro-specific ubiquitinated substrate incubated for 24 h s at room temperature under dark. Fluorescent reading was taken at 360/40 excitation and 460/40 nm emission using Synergy HT fluorescent plate reader. For dose-dependent studies, compounds were screened from concentrations of 0–100 µM. 10µL of 1% DMSO with enzyme and 50 µM of substrate served as the positive control. Wells with 50 µM of GRL0617 compounds (BPS Biosciences) served as specificity controls. Wells with only 1% DMSO and 50 µM of substrate served as blank. All the values were subtracted from blank values to calculate the percent activity of the enzymes.

### Cell viability assay

The cytotoxic effect of selected phytochemicals were carried out with Vero-E6 cells using PrestoBlue™ Cell Viability kit as described earlier (Lewis et al., 2022). Briefly, Vero-E6 cells (20,000 cells) were seeded overnight in 96 well plates. Then, 100 µL of complete media (EMEM+10% FBS+1%Pen/Strep) was added to refresh the cells along with 50, 100, and 200 µM selected phytochemicals, and incubation was continued for 24, 48, and 72 hs at 37°C respectively. Cytotoxicity detection regent (PrestoBlue solution) was added and incubated for additional 1h at 37°C. The absorbance was taken at 530/25 excitation and 590/35 nm emission using Bio-Tek Synergy HT fluorescent plate reader.

### Preparation of phytochemicals and PLpro protein for computational studies

Molecular Operating Environment (MOE) 2020.09 was used to conduct molecular docking *in silico* studies using the Amber10:EHT forcefield. The crystal structure of SARS-CoV-2 PLpro was retrieved from the protein data bank (www.rcsb.org) with PDB format (ID: 7CMD and was prepared with the MOE QuickPrep application under default settings. The selected phytochemicals were prepared with the program ChemDraw Professional, Version 10, Cambridge Soft.

Integrated Computer-Aided Molecular design computing method MOE was used to dock both phytochemicals with PLpro. All phytochemicals were examined individually and refined with the Triangle Matcher placement method and induced fit protocol. The docked molecules were scored with the GBVI/WSA dG scoring function.

### Statistical analysis and reproducibility

Statistical analysis was carried out using one-way analysis of variance (ANOVA) with Bonferroni’s Multiple Comparison test with 99.9% confidence intervals and represented as the mean ± SEM. Two-way ANOVA was conducted with Dunnett’s post-test to compare the grouped data. *p* values *p* < .05 considered statistically significant. Non-linear regression (curve fit) with four variable dose vs*.* inhibition was performed to calculate the IC_50_ values. GraphPad Prism (version 8; La Jolla, CA, United States) was used for statistical analysis. Four individual experiments were performed and triplicates are included in each experiment for reproducibility.

## Results

### Inhibition of SARS-CoV-2 PLpro proteolytic activity by phytochemicals

**FIGURE 1:**
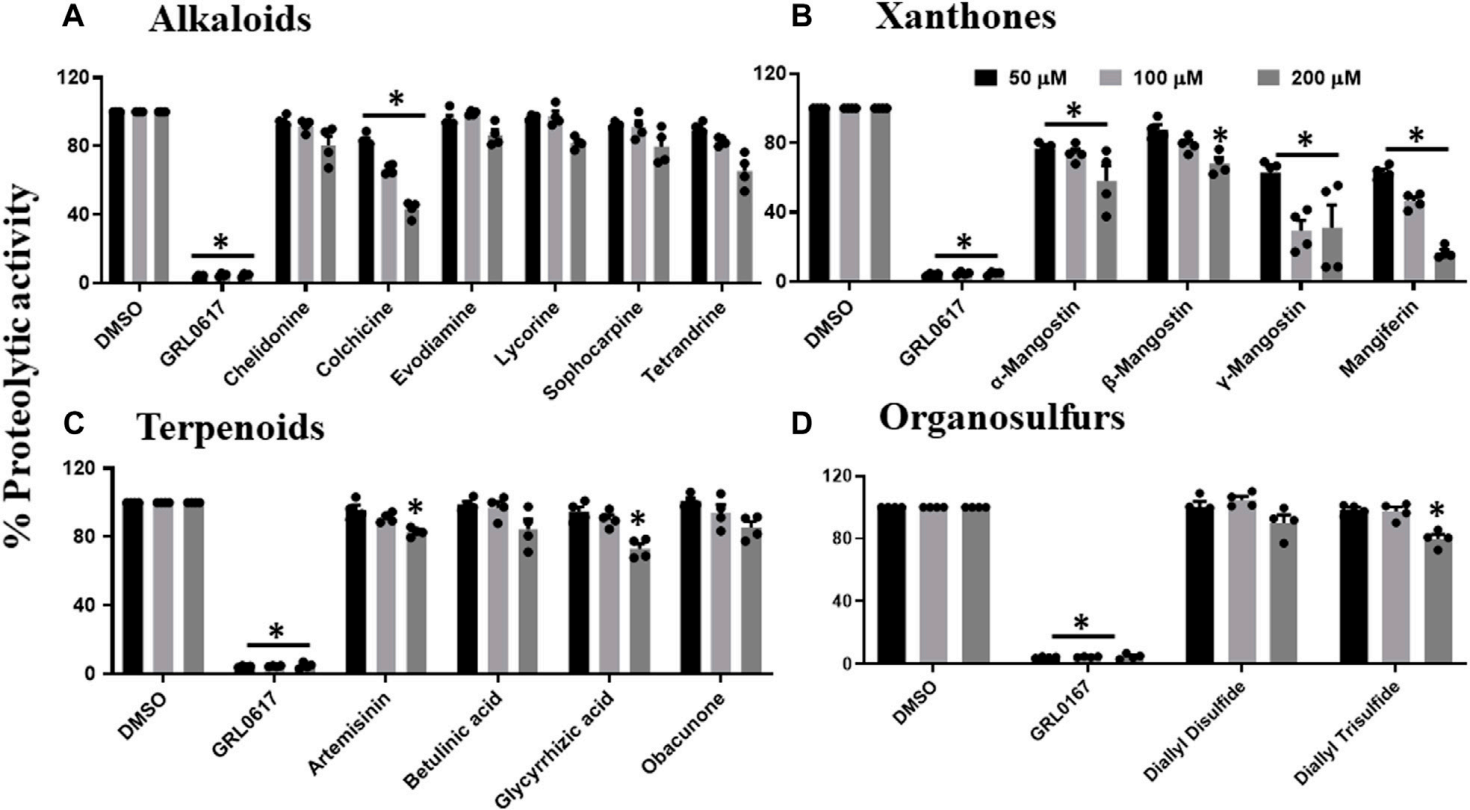
Inhibition of proteolytic activity of SARS-CoV-2 PLpro enzyme by selected alkaloids **(A)**, xanthones **(B)**, terpenoids **(C)** and organosulfur molecules **(D)**. Selected alkaloids, xanthones, terpenoids, and organosulfur compounds were screened for their inhibitory activity against the SARS-CoV-2 PLpro enzyme as described under Materials and Methods. The fluorescence intensity was used to calculate the percent proteolytic activity considering DMSO treated control as 100% activity. Blank values were subtracted before calculating the percent activity. Representatives of four individual experiments (n = 4) with triplicate values were presented graphically and analyzed using GraphPad Prism 8. *p* <.05 considered as statistically significant compared to the DMSO control.

Since phytochemicals are known to inhibit viral replication or viral entry, we performed the high throughput screening of a series of phytochemicals, selected based on the literature review, towards SARS-CoV-2 PLpro proteolytic activity (Supplementary Table S2). Our data suggest that nine out of the 53 selected phytochemicals have the potential to inhibit SARS-CoV-2 PLpro proteolytic activity at least by 50% or lower when compared to untreated samples. Figure 1A reveals that only colchicine out of all the tested alkaloids such as chelidonine, evodiamine, lycorine, sophocarpine and tetrandrine, could inhibit PLpro proteolytic activity. Colchicine was able to inhibit around 50% of the proteolytic activity at 200 μM. The four xanthone compounds tested were α, β, and γ-mangostin and mangiferin. Only γ-mangostin and mangiferin exhibited more than 50% inhibitory activity at the higher concentration (200 μM) against SARS-CoV-2 PLpro proteolytic activity (Figure 1B). We observed that the terpenoids such as artemisinin, betulinic acid, glycyrrhizic acid, obacunone, and organosulfur compounds, including diallyl disulfide and diallyl trisulfide, did not show inhibitory activity towards SARS-CoV-2 PLpro proteolytic activity at all the tested concentrations (Figure 1D).

**FIGURE 2:**
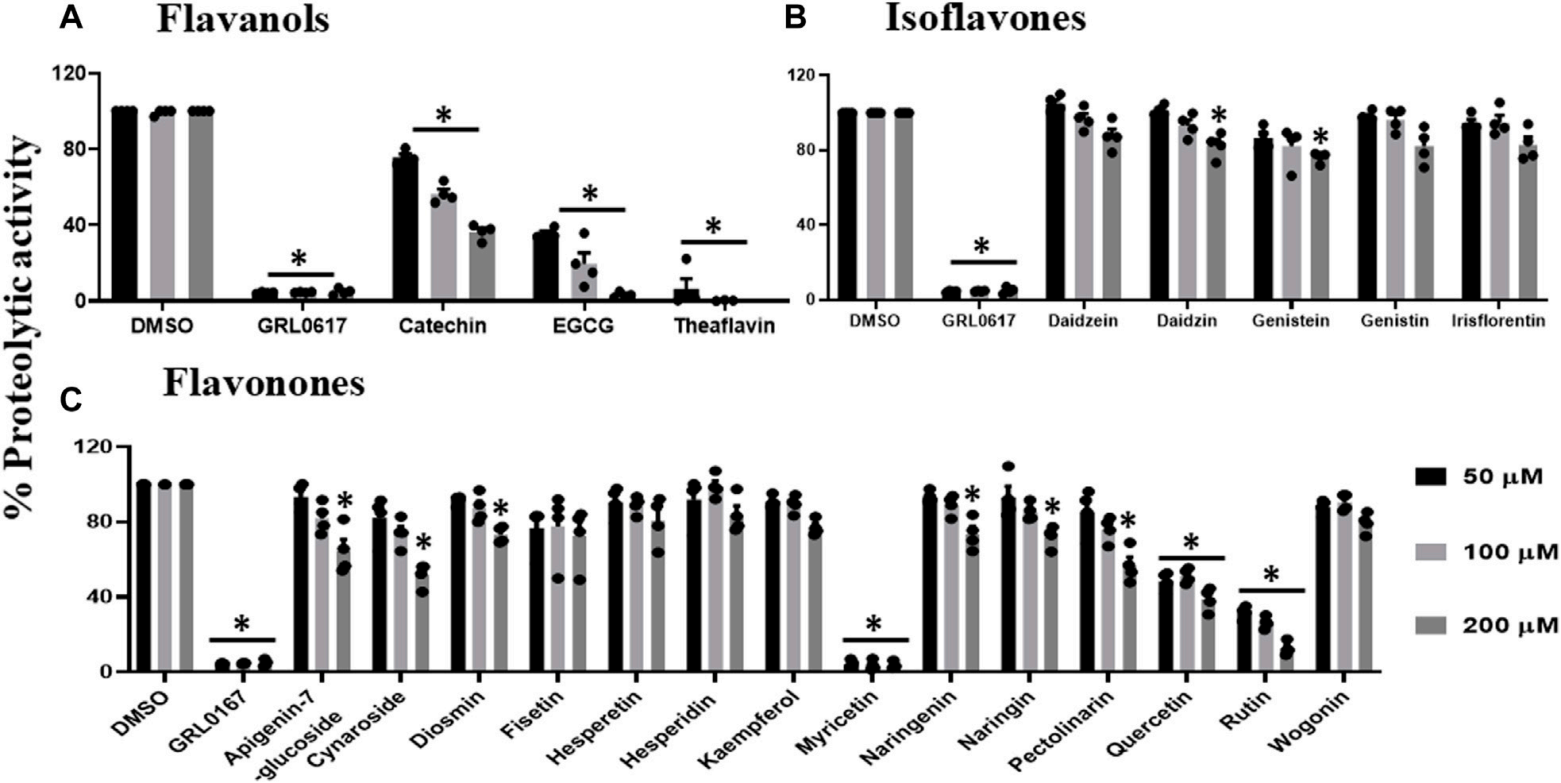
Inhibition of proteolytic activity of SARS-CoV-2 PLpro enzyme by selected flavanols **(A)**, isoflavones **(B)** and flavanones **(C)**. Selected flavanols, isoflavones and flavanones were screened for their inhibitory activity against the SARS-CoV-2 PLpro enzyme as described under Materials and Methods. The fluorescence intensity was used to calculate the percent proteolytic activity considering DMSO treated control as 100% activity. Blank values were subtracted before calculating the percent activity. Representatives of four individual experiments (n = 4) with triplicate values were presented graphically and analyzed using GraphPad Prism 8. *p*-values <.05 considered as statistically significant compared to the DMSO control.

**FIGURE 3:**
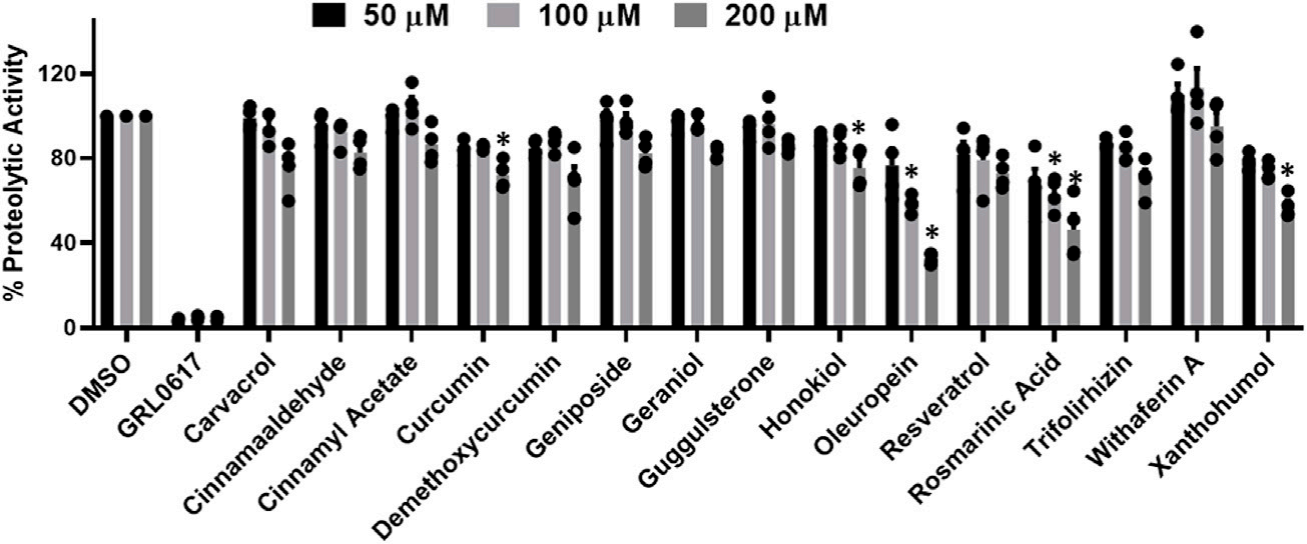
Inhibition of proteolytic activity of SARS-CoV-2 PLpro enzyme by selected miscellaneous phytochemicals. Miscellaneous phytochemicals were selected and screened for their inhibitory activity against the SARS-CoV-2 PLpro enzyme as described under Materials and Methods. The fluorescence intensity was used to calculate the percent proteolytic activity considering DMSO treated control as 100% activity. Blank values were subtracted before calculating the percent activity. Representatives of four individual experiments (n = 4) with triplicate values were presented graphically and analyzed using GraphPad Prism 8. *p*-values <.05 considered as statistically significant compared to the DMSO control.

All flavanols (Figure 2A), including catechin, EGCG, and theaflavin, exhibited a significant decrease in proteolytic activity. The inhibitory effect of catechin was 25%, 44%, and 64%, EGCG was 65%, 81%, and 97%, and theaflavin had an average of 94%, 100% at 50, 100, and 200 μM. We observed that none of the tested isoflavone compounds such as daidzein, daidzin, genistein, genistin, and isoflorentin demonstrated an inhibitory effect against PLpro proteolytic activity at the tested doses (Figure 2B). As shown in Figure 2C, flavanones such as myricetin inhibited 96% at as low as 50 μM. Interestingly, quercetin inhibited 50% at 50μM, and its inhibitory effect did not alter at higher concentration of 200 μM. Rutin exhibited 70% inhibitory activity at 50 and 100 μM and 88% at 200 μM. Non-etheless, none of the miscellaneous agents (Figure 3) except oleuropein and rosmarinic acid exhibited significant inhibition of the proteolytic activity of SARS-CoV-2 PLpro (60% and 50% at 200 μM).

### Inhibition of SARS-CoV-2 PLpro deubiquitinase activity by selected phytochemicals

**FIGURE 4:**
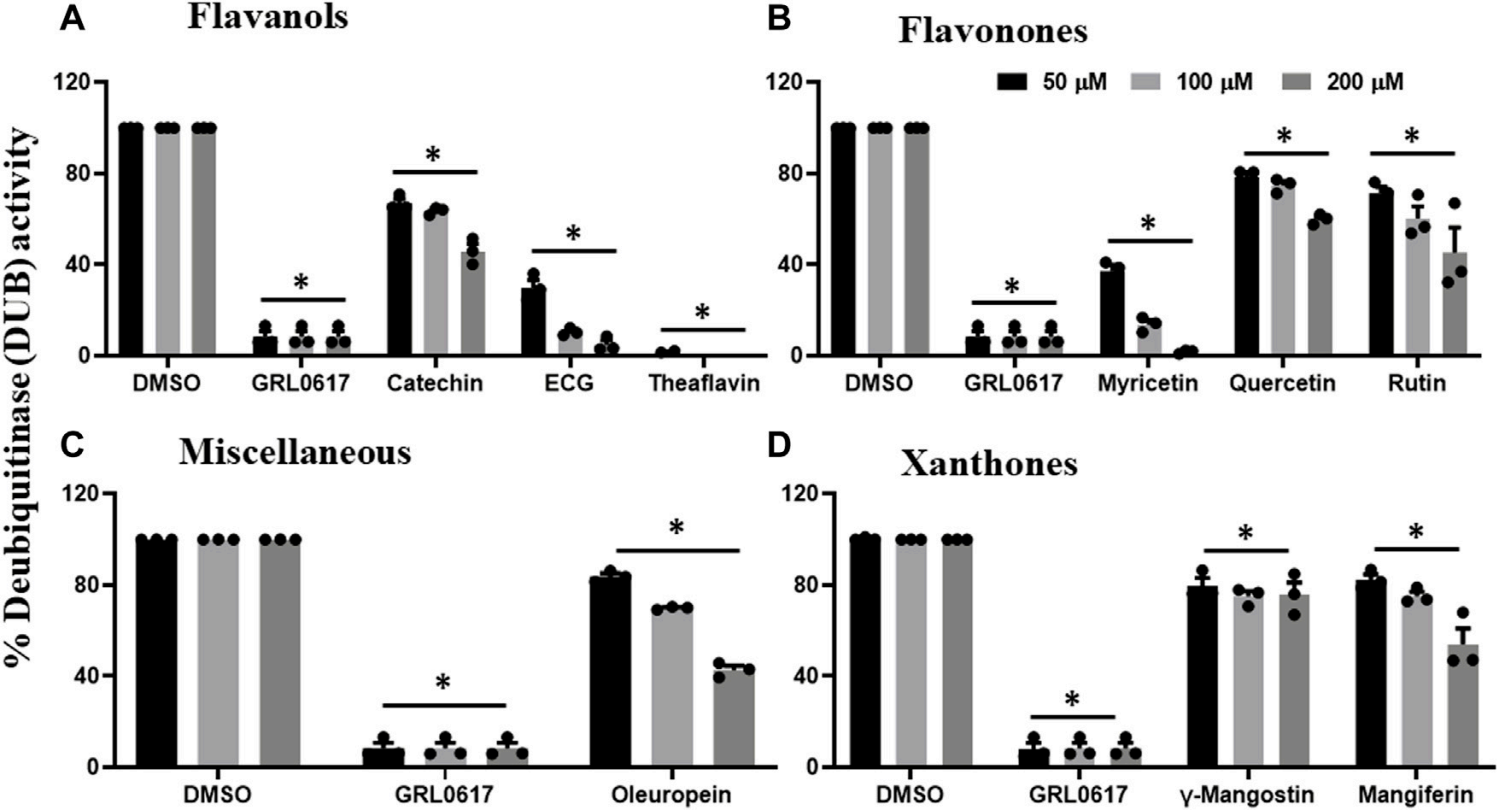
Selected phytochemicals Flavanols **(A)**, Flavonones **(B)**, Miscelleneous Drugs **(C)**, and Xanthones **(D)** that exhibited inhibitory effect against deubiquitinase (DUB) activity of SARS-CoV-2 PLpro enzyme. Phytochemicals that inhibited the proteolytic activity of SARS-CoV-2 PLpro enzyme were selected and screened for their inhibitory effect towards DUB activity as described under Materials and Methods. The fluorescence intensity was used to calculate the percent DUB activity considering DMSO treated control as 100% activity. Blank values were subtracted before calculating the percent activity. Representatives of four individual experiments (n = 4) with triplicate values were presented graphically and analyzed using GraphPad Prism 8. *p*-values <.05 considered as statistically significant compared to the DMSO control.

Previous data revealed that catechin, EGCG, theaflavin, myricetin, quercetin, rutin, oleuropein, γ-mangostin, and mangiferin inhibited proteolytic activity of PLpro enzyme, and therefore, we performed DUB activity in the presence of these inhibitory phytochemicals. As shown in Figure 4A, flavanols such as catechin inhibited the DUB activity by 30%, 40%, and 50%, whereas, EGCG by 70%, 90%, and 95%, at 50, 100, and 200 μM, respectively. Interestingly, theaflavin exhibited complete inhibition of DUB activity at concentration as low as 50 μM. Flavonones (myricetin, quercetin, and rutin) were selected to test for potential inhibitory activity against SARS-CoV-2 PLpro’s deubiquitinase activity. Figure 4B suggests that myricetin inhibited DUB activity by 60%, 90%, and 100% at 50, 100, and 200 μM, respectively, whereas quercetin and rutin exhibited minimal inhibitory (20%–40%) activity against DUB activity of PLpro at the tested doses. Oleuropein was the only polyphenol compound classified as miscelleanous agent that exhibited partial inhibitory activity (50% at 200 μM) against SARS-CoV-2 PLpro’s DUB activity (Figure 4C). Interestingly, xanthones such as γ -mangostin (50%) and mangiferin exhibited (70%) inhibitory activity towards the proteolytic activity of the PLpro enzyme (Figure 1B), but we observed very minimal DUB inhibitory activity (10%–30%) even at 200 μM concentration (Figure 4B). At present we do not know the reason for the difference in the inhibitory activity of γ-mangostin and mangiferin against proteolytic and DUB activity of SARS CoV-2 PLpro enzyme. The higher inhibitory effect of mangiferin towards proteolytic activity of PLpro might be due to the presence glycosidic unit compared to γ -mangostin, as we observed with Aloin-A and B (29).

### Dose-dependent inhibition of proteolytic and DUB activity of SARS-CoV-2 PLpro enzymatic activity

**FIGURE 5:**
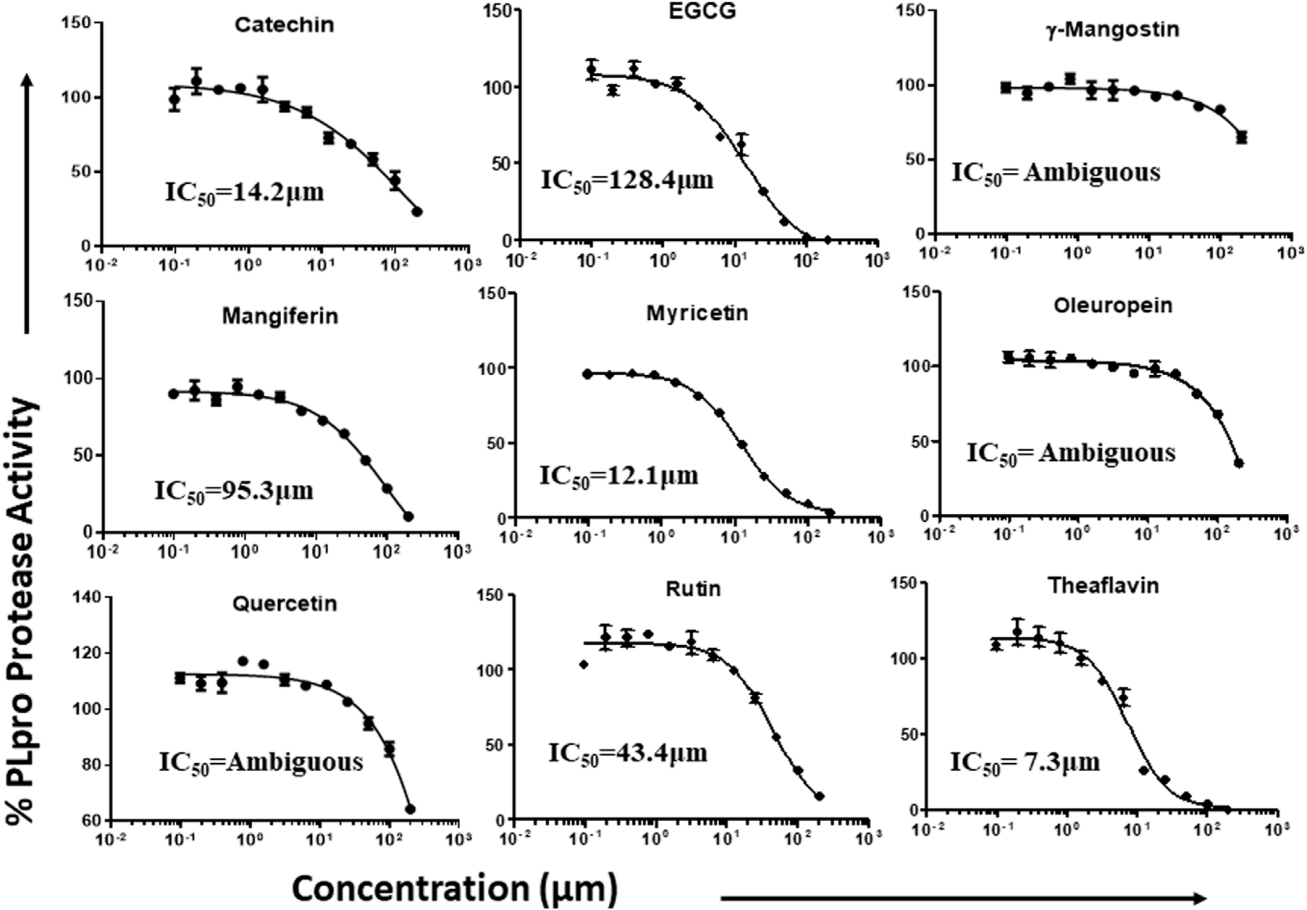
Dose-dependent inhibition of SARS-CoV-2 PLpro proteolytic activity by EGCG, catechin, myricetin, mangiferin, and γ-mangostin, oleuropein, quercetin, rutin, and theaflavin: The phytochemicals that exhibited at least 50% inhibition of proteolytic activity of PLpro enzyme were selected and screened for their dose-dependent inhibitory activity as described under Materials and Methods. The fluorescence intensity was used to calculate the percent enzymatic activity considering DMSO treated control as 100% activity. Blank values were subtracted before calculating the percent activity. Representatives of four individual experiments (n = 4) with triplicate values were analyzed using GraphPad Prism8 and presented graphically. IC50 values were calculated using non-linear regression (curve fit) with four variable dose vs*.* inhibition by GraphPad Prism8.

**FIGURE 6:**
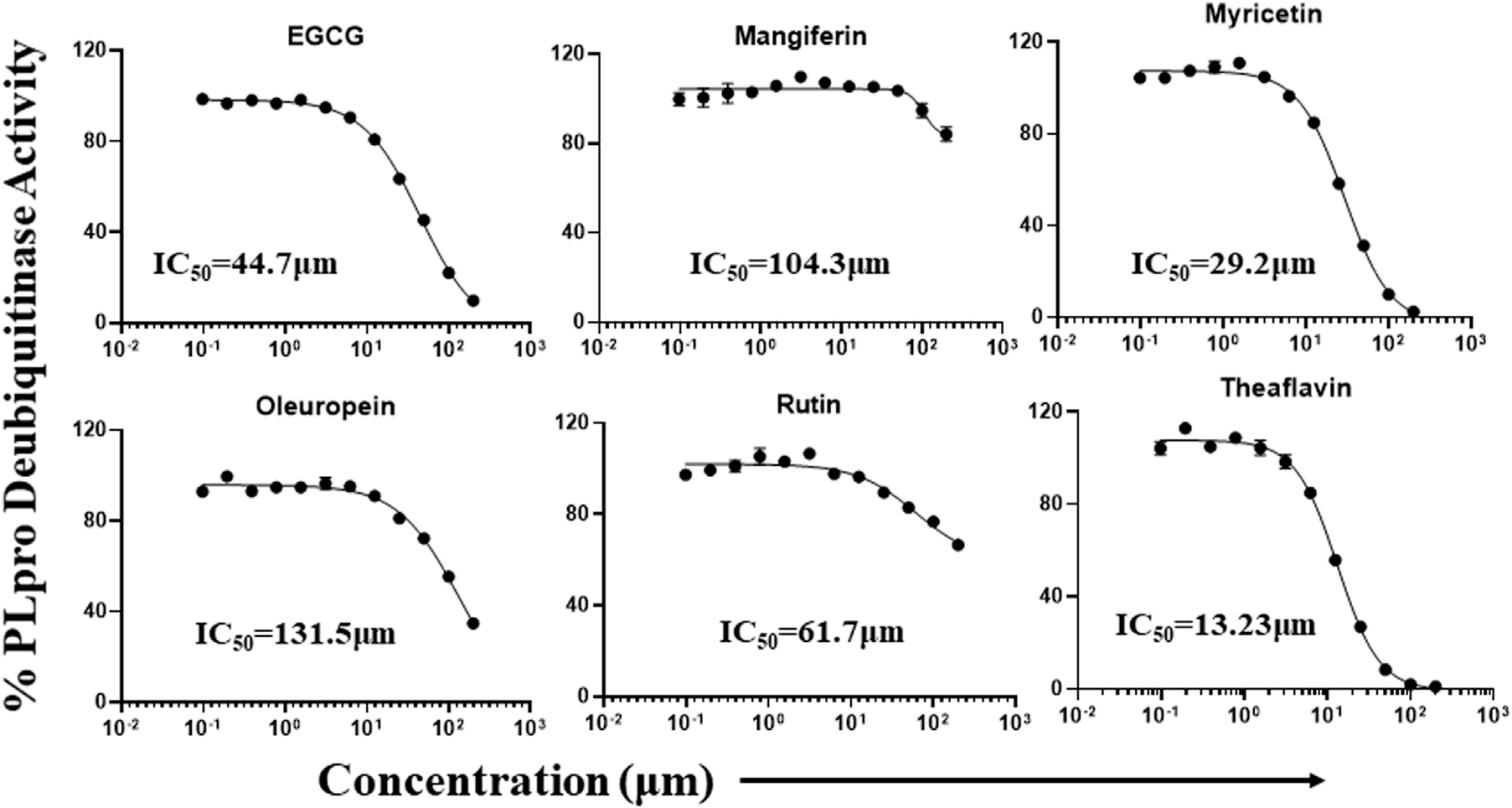
Dose-dependent inhibition of SARS-CoV-2 PLpro DUB activity by EGCG, mangiferin, myricetin, oleuropein, rutin, and theaflavin: The phytochemicals that exhibited at least 50% inhibition of DUB activity of PLpro enzyme were selected and screened for their dose-dependent inhibitory activity as described under Martials and Methods. The fluorescence intensity was used to calculate the percent enzymatic activity considering DMSO treated control as 100% activity. Blank values were subtracted before calculating the percent activity. Representatives of four individual experiments (n = 4) with triplicate values were analyzed using GraphPad Prism8 and presented graphically. IC50 values were calculated using non-linear regression (curve fit) with four variable dose vs*.* inhibition by GraphPad Prism8.

Phytochemicals that exhibited more than 50% inhibitory activity against proteolytic and DUB activity of SARS-CoV-2 PLpro enzyme were tested for dose-dependent inhibitory effects to determine the concentration required to inhibit the 50% of enzymatic activity (IC_50_). Here we represent the IC_50_ values of the selected phytochemicals that exhibited inhibitory activity against the PLpro enzyme. As shown in the Figure 5, catechin, EGCG, myricetin, mangiferin, rutin, and theaflavin exhibited IC_50_ values of 14.2, 128.4, 12.1, 43.4, 95.3, and 7.3 μM, respectively towards PLpro proteolytic activity. However, the IC_50_ values γ-mangostin, oleuropein, and quercetin are ambiguous. We observed that EGCG, mangiferin, myricetin, oleuropein, rutin, and theaflavin have IC_50_ values of 44.7, 104.3, 29.2, 131.5, 61.7, and 13.2 μM, respectively for DUB activity (Figure 6).

Furthermore, we investigated the cytotoxic effect of EGCG, mangiferin, myricetin, oleuropein, rutin, and theaflavin since they exhibited inhibitory activity for both proteolytic and DUB activity of PLpro. Out of the six phytochemicals tested, four of them did not have cytotoxic effect on Vero-E6 for 24, 48, and 72 h s at 50, 100, and 200 µM, respectively. However, we observed minimal cytotoxic effect (20%–30%) from myricetin and oleuropein after 48 hs (Supplementary Figure S1). We selected Vero-E6 cell line because of its sensitivity towards SARS-CoV-2 infection (Ren et al., 2006; Asrani et al., 2021). Although selected phytochemicals exhibited minimal cytotoxic effects, it is possible that the cytotoxic effects of phytochemicals might be altered in the virus infected cells, therefore it is warranted that the effective concentration of these phytochemicals may change during viral challenge studies.

### Structural interaction of phytochemicals with SARS-CoV-2 PLpro

The structure of SARS-CoV-2 PLpro is divided into four main sub-domains, the N-terminal Ubiquitin-like domain, α-helical thumb domain, β-stranded finger domain and the palm domain. The structure of ubiquitin-specific proteases deubiquitinating enzyme (DUB) shares a low homology (10%) (Osipiuk et al., 2021) with that of SARS-CoV-2 PLpro. The fingers subdomain is made of six β-strands and two α-helices, whereas, the thumb comprises six α-helices and a small β-hairpin. The palm subdomain is comprised of six β-strands. The proteolytic and DUB sites in SARS-CoV-2 PLpro are independent of each other implying two possible activities of PLpro. The interface of palm and thumb subdomains is the location for conventional catalytic triad Cys^111^-His^272^-Asp^286^. In addition to the catalytic triad, three additional residues play an important role in the enzymatic activity of SARS-CoV-2 PLpro: β-turn/loop (Glu^266^ -Gly^271^) which closes upon substrate and/or inhibitor binding is found adjacent to the active site. The Tyr^268^ part of the (Glu^266^ -Gly^271^) plays a critical role in the proteolytic activity of SARS-COV-2 PLpro and the Glu^167^ in SARS-COV-2 PLpro plays an important role in ubiquitin core recognition. The mutation of Tyr^268^ has shown to interfere with the proteolytic activity of SARS-COV-2 PLpro and the mutations of Glu^167^causes a significant loss of DUB activity (Osipiuk et al., 2021). Any molecule which forms hydrogen bond with Tyr^268^ or Glu^167^ will interfere with the proteolytic and the DUB activity of the SARS-CoV-2 PLpro, respectively and hence will show inhibitory activity.

**FIGURE 7:**
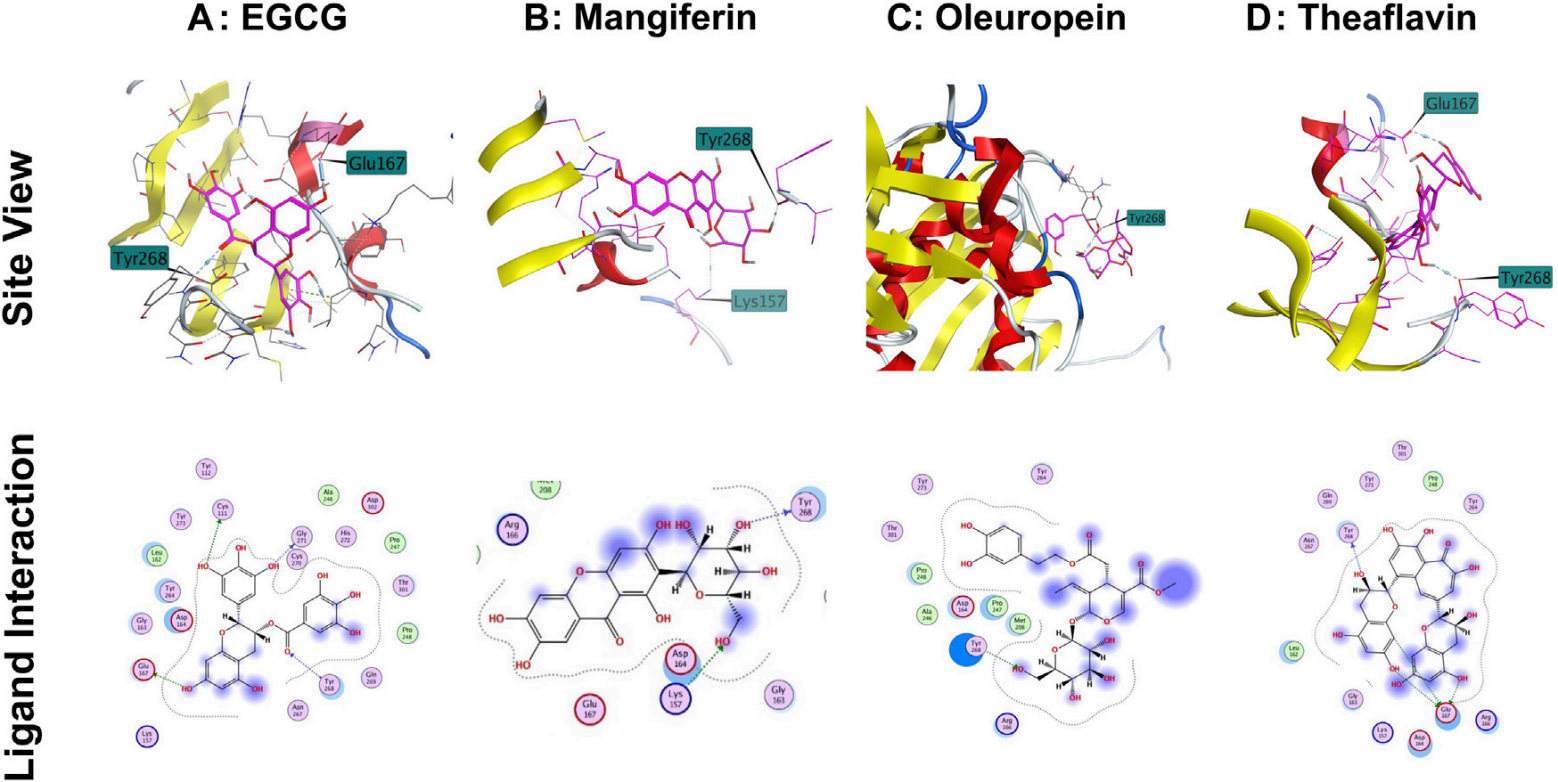
Interaction of selected phytochemicals with SARS-CoV-2 PLpro. **(A)** Interaction of EGCG with Glu^167^, Cys^111^ and Tyr^268^ of PLpro through hydrogen bonding. **(B)** Interaction of mangiferin with Tyr^268^ and Lys^157^ of PLpro through hydrogen bonding. **(C)** Interaction of oleuropein with Tyr^286^ of PLpro through hydrogen bonding. **(D)** Interaction of Theaflavin with Try^268^ and Glu^167^ of PLpro through hydrogen bonding. Site view and ligand interaction maps were presented to illustrate the hydrogen bonding.

**FIGURE 8:**
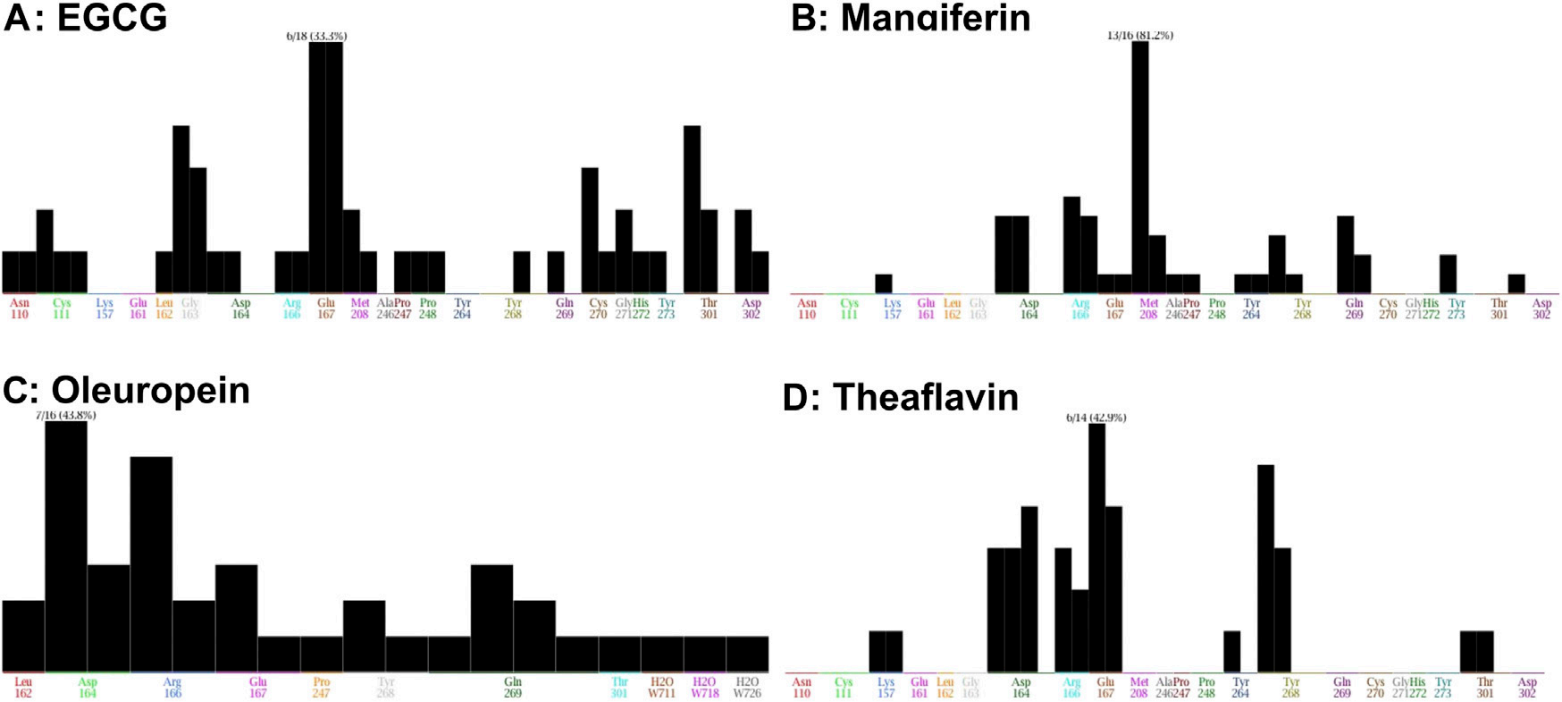
Fingerprinting population map of SARS-CoV-2 PLpro-ligand interaction. **(A)** EGCG shows a stronger interaction strength with Glu^167^ than Tyr^268^. **(B)** Mangiferin demonstrates a stronger interaction strength with Tyr^268^. **(C)** Oleuropein shows a weak interaction with Tyr^268^. **(D)** Theaflavin shows a weaker interaction with Tyr^268^ than Glu^167^ of PLpro but this interaction is stronger than EGCG, Mangiferin, and Oleuropein. The interaction of the molecules with specific amino acids of PLpro enzyme are represented as black bar.

The interaction of EGCG, mangiferin, myricetin, oleuropein, theaflavin, and rutin to the ligand site of GRL-0617, in the SARS-COV-2 PLpro (PDBID: 7cmd) was analyzed using MOE software. Molecular docking studies of EGCG, mangiferin, myricetin, oleuropein, theaflavin, and rutin with PLpro showed that myricetin and rutin did not interact with the catalytic site of PLpro enzyme and hence we were not able to obtain any docking studies for them and these molecules are not discussed further in molecular docking studies. The molecular docking studies resulted in 15–20 orientations each for the rest of the ligands used in the docking studies. The analysis of protein-ligand interaction between the SARS-CoV-2 PLpro enzyme and EGCG, and theaflavin showed a strong interaction with Try^268^ and Gln^167^ whereas, oleuropein and mangiferin interacted with Try^268^ (Figures 7A–D). As mentioned earlier, Glu^167^ plays an important role in the deubiquitination of the enzyme molecular modeling predicted that EGCG, and theaflavin significantly impair the DUB activity of SARS-CoV-2 PLpro. The ligand-protein fingerprint scan of mangiferin, EGCG, theaflavin, and oleuropein (Figures 8A–D) also shows that these four ligands show interaction with Glu^167^ and Tyr^268^, however mangiferin and oleuropein showed very weak interaction with Glu^167^ as compared to EGCG, and theaflavin which is reflected in their low IC_50_ values for DUB activity.

## Discussion

The outbreak of novel severe acute respiratory syndrome coronavirus-2 (SARS-CoV-2), causing the disease known as COVID-19, is a significant threat to public health globally because of the high rate of infection with the new emerging variants of concerns (Morens and Fauci, 2020; Wang and Yang, 2022c; Tiecco et al., 2022). Although global vaccination is currently ongoing, with the new mutations emerging, there is a greater need to develop therapeutics to protect high-risk populations, especially pregnant women, children, and people living with comorbidity. Currently, only a few antivirals (Paxlovid and Lagevrio) have been approved by the FDA for emergency use to treat COVID-19, however, rebound, mutated, commercialized antivirals remain expensive, and the mutagenic potential of these small molecule antivirals in human cells is challenging their effectiveness (Saravolatz et al., 2022). Previous studies suggested that viral replicative enzymes such as proteases are essential for viral replication (de Leuw and Stephan, 2018), and therefore, may serve as drug targets. The PLpro cleaves the newly generated PP chain auto-proteolytically to generate 3 NSPs required for the viral replication. Besides the protease activity, SARS-CoV-2 PLpro exhibits DUB activity responsible for inhibiting the host’s antiviral immune response (Mody et al., 2021; Lewis et al., 2022). Thus, PLpro serves as a drug target to inhibit viral replication and suppress the cytokine storm during SARS-CoV-2 infection.

Our data suggests that EGCG, myricetin, mangiferin, oleuropein, rutin, and theaflavin have the potential to inhibit SARS-CoV-2 PLpro proteolytic and DUB activity, whereas catechin, γ-mangostin and quercetin inhibits only proteolytic activity. This study indicates that the above-mentioned phytochemicals are potential inhibitors against SARS-CoV-2 PLpro proteolytic and deubiquitinase activity.

EGCG is an antioxidant commonly found in green and black tea and is the most potent catechin derivative for antitumor activity and disrupting molecular signaling pathways in breast, pancreatic, prostate, lung, and stomach cancers based on *in vitro* and *in vivo* studies (Chourasia et al., 2021). EGCG also demonstrated antiviral properties in retroviruses such as the Zika virus, influenza A, and Chikungunya virus (Carneiro et al., 2016; Lu et al., 2017; Mou et al., 2020). The anti-SARS-CoV-2 effects of EGCG were reported to be mediated through the inhibition of 3CLpro enzyme (Jang et al., 2020). Mangiferin is a C-glucosyl xanthone (2-β-D-glucopyranosyl-1,3,6,7-tetrahydroxy-9H-xanthan-9-one) and a significant component in mango peel and seed with many valuable properties such as antioxidant, anti-microbial, anti-diabetic, anti-allergic, anticancer, hypocholesterolemic, and immunomodulatory effects (Imran et al., 2017). It is also known to inhibit the activation of peroxisome proliferator-activated receptors (Imran et al., 2017). Mangiferin is also known to inhibit the type-I herpes simplex virus (HSV-I) replication in a cell culture model (Imran et al., 2017). Myricetin is a 3, 5, 7, 3′, 4′, 5′-hexahydroxyflavone abundantly found in fruits, vegetables, tea, and some medicinal plants; however, its primary source is the Chinese bayberry (Zhang et al., 2015; Semwal et al., 2016). Myricetin draws much consideration because of its several health-beneficial effects: antioxidant, anti-inflammatory, anti-diabetic, anti-Alzheimer, anti-cancer, anti-bacterial, anti-microbial, and anti-viral (Semwal et al., 2016). Its antioxidant activity allows it to serve as a reactive oxygen species scavenger (Semwal et al., 2016). Recent study suggest that myricetin inhibits the SARS-CoV-2 3CLpro enzyme (Xiao et al., 2021). Oleuropein is the primary phenolic compound derived from *Olea europaea*, which also produces olive oil and possesses many pharmacological properties such as antioxidant, anti-inflammatory, anti-atherogenic, anti-cancer, antimicrobial, and antiviral (Omar, 2010). Several epidemiological and clinical studies suggest that theaflavin from black tea has remarkable pharmacological properties, including anti-inflammatory, antioxidant, anti-cancer, anti-obesity, anti-osteoporotic, anti-microbial, and anti-viral effects (Shan et al., 2021). Recent *in vitro* studies suggest that theaflavin inhibits the SARS-CoV-2 3CLpro enzyme (Jang et al., 2020). Rutin is a flavonoid derived from the plant *Ruta graveolens* and is commonly found in teas, apples, and buckwheat. Rutin is commonly known as vitamin P. Rutin has multiple pharmacological activities on different body systems, making it a solid therapeutic phytochemical to fight against several pathological conditions. Additionally, rutin also possesses antiviral activity against retroviruses and viruses such as herpes and hepatitis C and B (Ganeshpurkar and Saluja, 2017).

In conclusion, among the 53 phytochemicals tested, only EGCG, mangiferin, myricetin, oleuropein, theaflavin, and rutin effectively inhibited both proteolytic and DUB activities of PLpro enzyme. Additionally, EGCG, myricetin and theaflavin are known to inhibit 3CLpro, a main protease of SARS-CoV-2 (Jang et al., 2020; Xiao et al., 2021). Our data suggest that EGCG, myricetin and theaflavin also inhibits the SARS-CoV-2 PLpro enzymatic activity, thus providing the evidence of dual target of these molecules to inhibit the SARS-CoV-2 replication. The overall data suggests that the phytochemicals mentioned above have strong potential as antiviral drug candidates to inhibit the SARS-CoV-2 replication and regulate cytokine storm prevalent in COVID-19 patients. Nevertheless, the therapeutic potential of these phytochemicals needs to be further validated by viral challenges and clinical studies to halt the viral spread of SARS-CoV-2 infection.

## Data Availability

The original contributions presented in the study are included in the article/Supplementary Material, further inquiries can be directed to the corresponding authors.
