# Supplementary material for: Inhibitory effect of phytochemicals towards SARS-CoV-2 papain like protease (PLpro) proteolytic and deubiquitinase activity
Source: Front Chem. 2023 Jan 12;10:1100460. doi: 10.3389/fchem.2022.1100460 (PMC9878345; doi:10.3389/fchem.2022.1100460)
Supplement: Supplementary file 1 [file DataSheet1.PDF]

Inhibitory effect of phytochemicals towards SARS-CoV-2 papain like protease (PLpro) proteolytic and deubiquitinase activity

Anasha Kawall[1], Devin S. M. Lewis[2], Avini Sharma[1], Krishna Chavada[3], Rahul Deshmukh,[4] Srujana Rayalam[3], Vicky Mody[3], Shashidharamurthy Taval[3]

[1]Department of Biomedical Sciences, Philadelphia College of Osteopathic Medicine – Georgia Campus, Suwanee, GA, USA.

[2]Division of Research, Philadelphia College of Osteopathic Medicine – Georgia Campus, Suwanee, GA, USA.

[3]Department of Pharmaceutical Sciences, School of Pharmacy, Philadelphia College of Osteopathic Medicine – Georgia Campus, Suwanee, GA, USA.

[4]Department of Pharmaceutical Sciences, College of Pharmacy, Rosalind Franklin University – North Chicago, IL, USA.

Running Title: SARS-CoV-2 PLpro inhibition by phytochemicals

Keywords: SARS-CoV-2, replication, PLpro, phytochemicals, natural compounds

***Corresponding Authors:**

Shashidharamurthy Taval, Ph.D.,
Associate Professor, Department of Pharmaceutical Sciences,
Philadelphia College of Osteopathic Medicine, School of Pharmacy,
Room 3031, 625 Old Peachtree Road, Suwanee, GA-30024,
Tel: 678-407-7373, Fax: 678-407-7347, Email: rangaiahsh@pcom.edu

Vicky Mody, Ph.D.,
Associate Professor, Department of Pharmaceutical Sciences,
Philadelphia College of Osteopathic Medicine, School of Pharmacy,
Room 3031, 625 Old Peachtree Road, Suwanee, GA-30024,
Tel: 678-407-7386, Fax: 678-407-7347, Email: vickymo@pcom.edu

Supplementary figure 1.

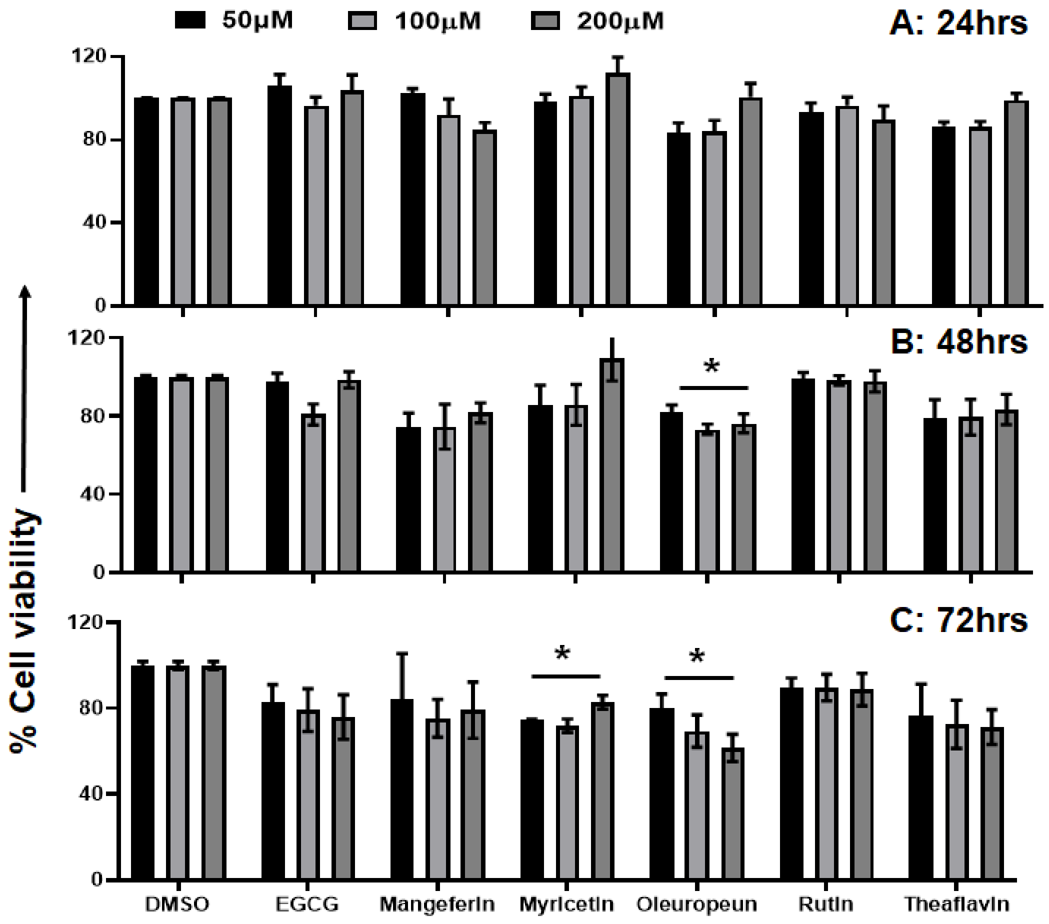

Cytotoxicity of selected phytochemicals in African green monkey kidney epithelial cell line Vero-E6. Cell viability assay was performed as described under materials and methods. EGCG, mangiferin, rutin, and theaflavin were not cytotoxic, but myricetin and oleuropein exhibited minimal cytotoxicity (20-30%) in Vero-E6 cells at the tested doses and time points. 0.1% DMSO treated cells were considered as positive control. Representative of two individual experiments (n=2) with triplicate values were presented graphically. Two-way ANOVA with Dunnett's Comparison post-test was used to calculate the statistical significance compared to the DMSO treated cells. <p.05 considered as statistically significant.

Supplementary table 1: List of tested phytochemicals with catalog number and manufacturers

Phytochemical	Manufacturer	Catalog number
**Flavonones**		
Naringenin	Cayman Chemicals	14173
Naringin	Cayman Chemicals	17923
Hesperidin (Hesperetin 7-rutinoside)	MedChemExpress	HY-15337
Hesperetin	MedChemExpress	HY-N0168
Apigenin 7-glucoside	Cayman Chemicals	26813
Pectolinarin	MedChemExpress	HY-N0314
Quercetin	Cayman Chemicals	10005169
Rutin	Cayman Chemicals	19868
Myricetin	Cayman Chemicals	10012600
Kaempferol	MedChemExpress	HY-14590
Wogonin	Cayman Chemicals	14248
Fisetin	MedChemExpress	HY-N0182
Cynaroside (Luteolin 7-glucoside)	MedChemExpress	HY-N0540
Diosmin	MedChemExpress	HY-N0178
**Isoflavones**		
Daidzein	Cayman Chemicals	10005166
Daidzin	Cayman Chemicals	13202
Genistein	Cayman Chemicals	10005167
Genistin	MedChemExpress	HY-N0595
Irisflorentin	MedChemExpress	HY-N0005
**Flavonones**		
Epigallocatechin gallate	MedChemExpress	HY-13653
Catechin	MedChemExpress	HY-N0898
Theaflavin	Cayman Chemicals	25129
**Terpenoids**		
Artemisinin	Cayman Chemicals	11816
Glycyrrhizic acid	Cayman Chemicals	11847
Betulinic acid	Cayman Chemicals	11686
Obacunone	MedChemExpress	HY-N0428
**Xanthones**		
α-Mangostin	MedChemExpress	HY-N0328
b-Mangostin	MedChemExpress	HY-N0941
ɣ-Mangostin	MedChemExpress	HY-N1957
Mangiferin	MedChemExpress	HY-N0290
**Organosulfur**		
Diallyl Disulfide	Cayman Chemicals	10012582
Diallyl Trisulfide	Cayman Chemicals	10012577

**Alkaloids**		
Colchicine	MedChemExpress	HY-16569
Chelidonine	MedChemExpress	HY-N2369
Evodiamine	MedChemExpress	HY-N0114
Lycorine	MedChemExpress	HY-N0288
Tetrandrine	MedChemExpress	HY-13764
Sophocarpine	Cayman Chemicals	29598
**Miscellaneous Flavonoids**		
Withaferin A	Cayman Chemicals	11352
Guggulsterone	Cayman Chemicals	10011296
Geniposide	MedChemExpress	HY-N0009
Rosmarinic Acid	Cayman Chemicals	70900
Resveratrol	Cayman Chemicals	14942
Trifolirhizin	MedChemExpress	HY-N0616
Oleuropein	Cayman Chemicals	21220
Xanthohumol	Cayman Chemicals	15399
Geraniol	Cayman Chemicals	23166
Curcumin	MedChemExpress	HY-N0005
Cinnamyl Acetate	Sigma-Aldrich	166170
Carvacrol	Cayman Chemicals	33753
Cinnamaldehyde	Sigma-Aldrich	W228613
Demethoxycurcumin	Cayman Chemicals	10961
Honokiol	MedChemExpress	HY-N0003

Supplementary table 2: Chemical structures of literature reviewed for phytochemicals with potential anti-SARS-CoV-2 effects *in-vitro* and *in-silico* studies

Phytochemical	Structure	Type of Study	Reference
**Flavonones**			
Apigenin 7-glucoside	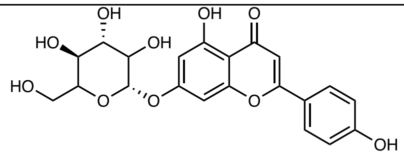	*In silico*	[1, 2]
Cynaroside (Luteolin 7-glucoside)	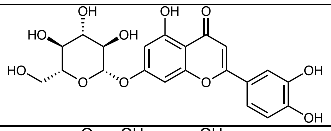	*In vitro*	[3]
Diosmin	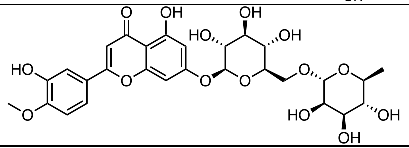	*In silico*	[1]
Fisetin	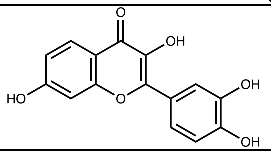	*In silico*	[2]
Hesperetin	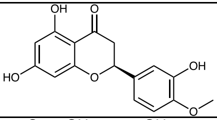	*In silico*	[2, 4]
Hesperidin	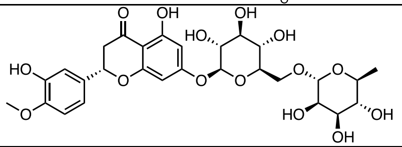	*In vitro*	[5]
Kaempferol	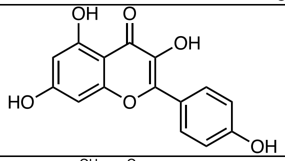	*In silico*	[1, 2]
Myricetin	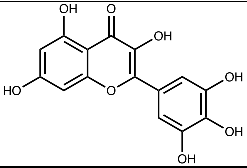	*In silico*	[1, 2]
Naringenin	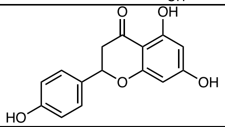	*In silico*	[1, 2]
Naringin	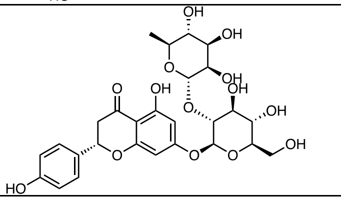	*In silico*	[1]

Pectolinarin	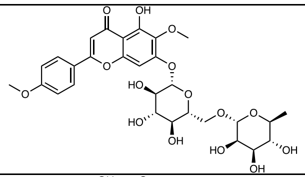	*In silico*	[1]
Quercetin	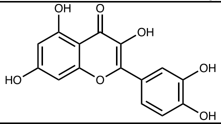	*In silico*	[1, 2]
Rutin	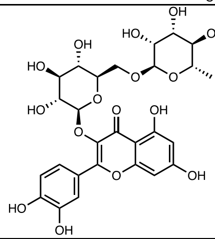	*In silico*	[1, 6]
Wogonin	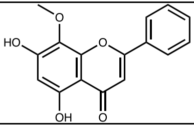	*In silico*	[7]
**Isoflavones**			
Daidzein	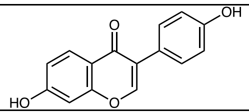	*In silico*	[8]
Daidzin	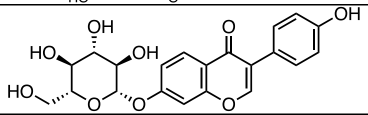	*In silico*	[9]
Genistein	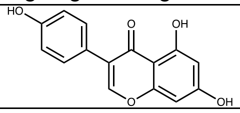	*In silico*	[6, 8]
Genistin	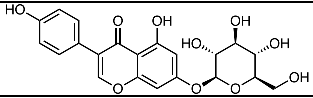	*In silico*	[10]
Irisflorentin	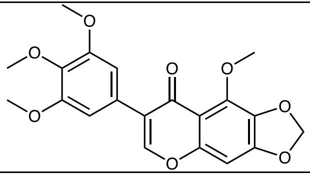	*In silico*	[11]
**Flavanols**			
Catechin	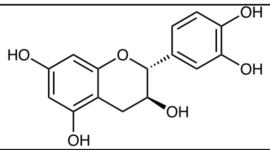	*In silico*	[6]
Epigallocatechin gallate	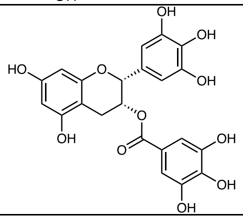	*In silico*<br>*In vitro*	[5, 6]

Theaflavin	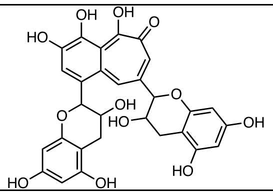	*In silico*	[1]
**Terpenoids**			
Artemisinin	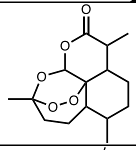	*In vitro*	[12]
Betulinic acid	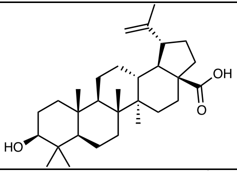	*In vitro*	[13]
Glycyrrhizic acid	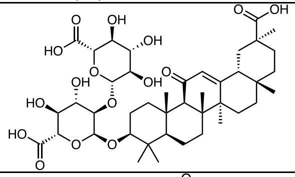	*In silico*	[14, 15]
Obacunone	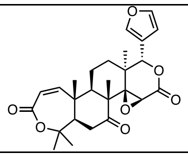	*In silico*	[14]
**Xanthones**			
α-Mangostin	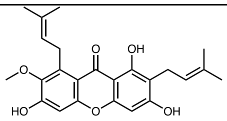	*In silico*	[16, 17]
b-Mangostin	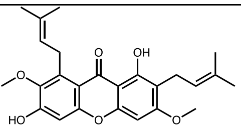	*In silico*	[17]
ɣ-Mangostin	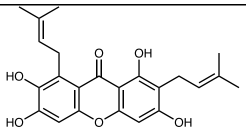	*In silico*	[17]
Mangiferin	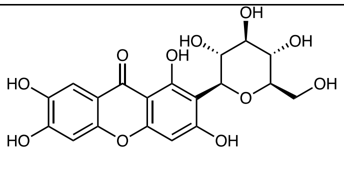	*In silico*	[18]
**Organosulfur**			
Diallyl Disulfide	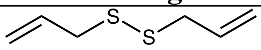	*In silico*	[19]

Diallyl Trisulfide	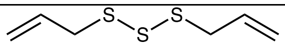	*In silico*	[19]
**Alkaloids**			
Colchicine	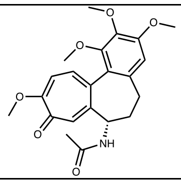	*In silico*	[20]
Chelidonine	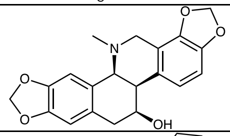	*In silico*	[21]
Evodiamine	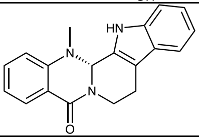	*In vitro*	[22]
Lycorine	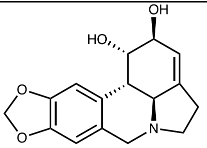	*In silico and in vitro*	[6]
Tetrandrine	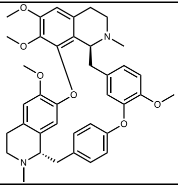	*In silico*	[6]
Sophocarpine	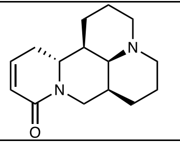	*In silico*	[6]
**Miscellaneous Flavonoids**			
Carvacrol	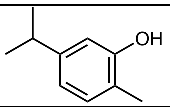	*In silico*	[8]
Cinnamaaldehyde	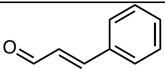	*In silico*	[8]
Cinnamyl Acetate	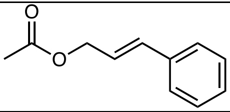	*In silico*	[23]
Curcumin	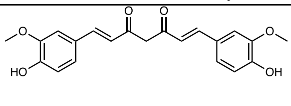	*In vitro*	[7] [24]
Demethoxycurcumin	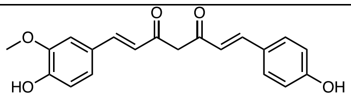	*In silico*	[25]

Geniposide	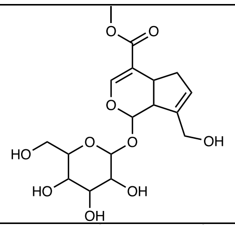	*In vitro*	[26]
Geraniol	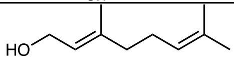	*In silico*	[26]
Guggulsterone	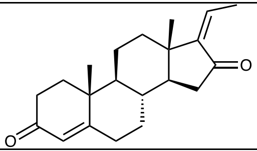	*In silico*	[27]
Honokiol	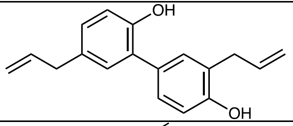	*In silico*	[7]
Oleuropein	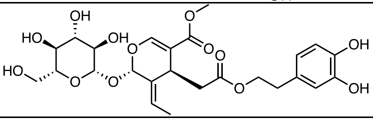	*In vitro*	[5]
Resveratrol	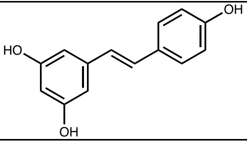	*In silico*	[6, 7]
Rosmarinic Acid	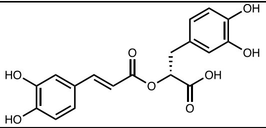	*In silico*	[11]
Trifolirhizin	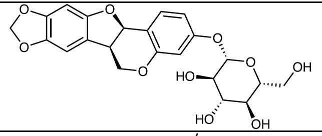	*In silico*	[28]
Withaferin A	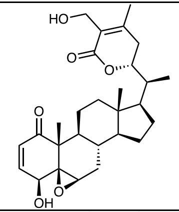	*In silico*	[29]
Xanthohumol	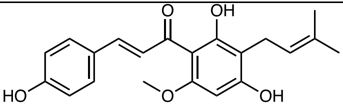	*In vitro*	[30]

References:

1. Murugesan, S., et al., *Targeting COVID-19 (SARS-CoV-2) main protease through active phytocompounds of ayurvedic medicinal plants - Emblica officinalis (Amla), Phyllanthus niruri Linn. (Bhumi Amla) and Tinospora cordifolia (Giloy) - A molecular docking and simulation study.* Comput Biol Med, 2021. **136**: p. 104683.
2. Vijayakumar, B.G., et al., *In silico pharmacokinetic and molecular docking studies of natural flavonoids and synthetic indole chalcones against essential proteins of SARS-CoV-2.* Eur J Pharmacol, 2020. **886**: p. 173448.
3. D. S. N. B. K. Prasanth, M.M., Vivek Chandramohanc, Gangadharappa Bhavyad, Atmakuri Lakshmana Raoe, Siva Prasad Pandaa, G. S. N. Koteswara Raof, Guntupalli Chakravarthia, Nayudu Tejag, Peddireddy Suguna Ranih, Gummadi Ashug, Chittiprolu Purnadurganjalie, Puvvala Akhile, Gorriputti Vedita Bhavanie, and Tirumalasetti Jaswitha, *In-silico strategies of some selected phytoconstituents from Melissa officinalis as SARS CoV-2 main protease and spike protein (COVID-19) inhibitors.* MOLECULAR SIMULATION, 2021. **46**(6): p. 457-470.
4. Cheng, F.J., et al., *Hesperidin Is a Potential Inhibitor against SARS-CoV-2 Infection.* Nutrients, 2021. **13**(8).
5. Aynur MK, C.M.A., Ihsan K, Ali Arslan, *Antiviral Effects of Some Flavonoids on SARS-CoV-2* Turkish Journal of Science and Health 2021. **2**(3): p. 24.34.
6. Gurung, A.B., et al., *Unravelling lead antiviral phytochemicals for the inhibition of SARS-CoV-2 M(pro) enzyme through in silico approach.* Life Sci, 2020. **255**: p. 117831.
7. Monjur AL, M.D., *Search for therapeutics against COVID 19 targeting SARS-CoV-2 papain-like protease: an in silico study.* Research Square, 2022.
8. Elfiky, A.A., *Natural products may interfere with SARS-CoV-2 attachment to the host cell.* J Biomol Struct Dyn, 2021. **39**(9): p. 3194-3203.
9. Huang C-W, H.H.-A., Tsai S-C, et al, *In Silico Target Analysis of Treatment for COVID-19 Using Huang-Lian-Shang-Qing-Wan, a Traditional Chinese Medicine Formula.* Natural Product Communications, 2021. **16**(10).
10. Harisna, A.H., et al., *In silico investigation of potential inhibitors to main protease and spike protein of SARS-CoV-2 in propolis.* Biochem Biophys Rep, 2021. **26**: p. 100969.
11. Yang, R., et al., *Chemical composition and pharmacological mechanism of Qingfei Paidu Decoction and Ma Xing Shi Gan Decoction against Coronavirus Disease 2019 (COVID-19): In silico and experimental study.* Pharmacol Res, 2020. **157**: p. 104820.
12. Cao, R., et al., *Anti-SARS-CoV-2 Potential of Artemisinins In Vitro.* ACS Infect Dis, 2020. **6**(9): p. 2524-2531.
13. Wen, C.C., et al., *Specific plant terpenoids and lignoids possess potent antiviral activities against severe acute respiratory syndrome coronavirus.* J Med Chem, 2007. **50**(17): p. 4087-95.
14. Vardhan, S. and S.K. Sahoo, *In silico ADMET and molecular docking study on searching potential inhibitors from limonoids and triterpenoids for COVID-19.* Comput Biol Med, 2020. **124**: p. 103936.
15. Patil, R., et al., *Computational and network pharmacology analysis of bioflavonoids as possible natural antiviral compounds in COVID-19.* Inform Med Unlocked, 2021. **22**: p. 100504.

16. Hidayat, S., et al., *The interaction of alpha-mangostin and its derivatives against main protease enzyme in COVID-19 using in silico methods.* J Adv Pharm Technol Res, 2021. **12**(3): p. 285-290.
17. ANM Ansori, V.K., AA Parikesit, FA Dian, RT Probojati, M Rebezov, P Scherbakov, P Burkov, G Zhdanova, A Mikhalev, Y Antonius, MRF Pratama, NI Sumantri, TH Sucipto, and R Zainul, *Bioactive Compounds from Mangosteen (Garcinia mangostana L.) as an Antiviral Agent via Dual Inhibitor Mechanism against SARSCoV- 2: An In Silico Approach.* Pharmacognosy Journal, 2022. **14**(1): p. 85-90.
18. Singh, R., et al., *Protease Inhibitory Effect of Natural Polyphenolic Compounds on SARS-CoV-2: An In Silico Study.* Molecules, 2020. **25**(20).
19. Aanouz, I., et al., *Moroccan Medicinal plants as inhibitors against SARS-CoV-2 main protease: Computational investigations.* J Biomol Struct Dyn, 2021. **39**(8): p. 2971-2979.
20. Ehab M. Mostafa, M.G., Mohammed M. Ghoneim, Shaimaa Hussein, Ahmed H. El-Ghorab, Mohamed A. Abdelgawad2, Arafa Musa., *Repurposing of FDA Approved Alkaloids as COVID 19 Inhibitors; in silico Studies.* Pharmacognosy Journal, 2021. **13**(1): p. 110-123.
21. Wang Z, W.D., Belecciu T, Eaves J, Bachmann M, *Phytochemical Drug Discovery for COVID-19 Using High-resolution Computational Docking and Machine Learning Assisted Binder Prediction.* ChemRxiv, 2022. **Version 2**.
22. Vidoni, C., et al., *Targeting autophagy with natural products to prevent SARS-CoV-2 infection.* J Tradit Complement Med, 2022. **12**(1): p. 55-68.
23. Kulkarni, S.A., et al., *Computational evaluation of major components from plant essential oils as potent inhibitors of SARS-CoV-2 spike protein.* J Mol Struct, 2020. **1221**: p. 128823.
24. Bormann, M., et al., *Turmeric Root and Its Bioactive Ingredient Curcumin Effectively Neutralize SARS-CoV-2 In Vitro.* Viruses, 2021. **13**(10).
25. Mulu, A., et al., *The impact of curcumin derived polyphenols on the structure and flexibility COVID-19 main protease binding pocket: a molecular dynamics simulation study.* PeerJ, 2021. **9**: p. e11590.
26. Umeoguaju, F.U., et al., *Plant-Derived Food Grade Substances (PDFGS) Active Against Respiratory Viruses: A Systematic Review of Non-clinical Studies.* Front Nutr, 2021. **8**: p. 606782.
27. Ali, F., et al., *Implication of in silico studies in the search for novel inhibitors against SARS-CoV-2.* Arch Pharm (Weinheim), 2022. **355**(5): p. e2100360.
28. ShuoWang, et al., *Chloroform extract from Sophora Tonkinensis Gagnep. inhibit proliferation, migration, invasion and promote apoptosis of nasopharyngeal carcinoma cells by silencing the PI3K/AKT/mTOR signaling pathway.* J Ethnopharmacol, 2021. **271**: p. 113879.
29. Kumar, V., et al., *Withanone and Withaferin-A are predicted to interact with transmembrane protease serine 2 (TMPRSS2) and block entry of SARS-CoV-2 into cells.* J Biomol Struct Dyn, 2022. **40**(1): p. 1-13.
30. Yuan, S., et al., *SARS-CoV-2 exploits host DGAT and ADRP for efficient replication.* Cell Discov, 2021. **7**(1): p. 100.
